# Hypoxia and Cellular Senescence, Emerging Toxic Mechanisms of Mycotoxins and Toxins: A New Understanding of the Negative Immune Regulations

**DOI:** 10.3390/toxins14120880

**Published:** 2022-12-16

**Authors:** Qinghua Wu, Li You, Wenda Wu, Xu Wang

**Affiliations:** 1 College of Life Science, Yangtze University, Jingzhou 434025, China; 2 Department of Chemistry, Faculty of Science, University of Hradec Králové, 50003 Hradec Králové, Czech Republic; 3 College of Physical Education and Health, Chongqing College of International Business and Economics, Chongqing 401520, China; 4 School of Food and Biological Engineering, Hefei University of Technology, Hefei 230009, China; 5 National Reference Laboratory of Veterinary Drug Residues (HZAU) and MAO Key Laboratory for Detection of Veterinary Drug Residues, Huazhong Agricultural University, Wuhan 430070, China

Mycotoxin contamination is an important issue that has plagued the world. Mycotoxins not only contaminate food and feed but, more importantly, can further cause poisoning in humans and animals through food and feed. Moreover, children seem to be more sensitive to mycotoxins [1]. The risk of exposure to the trichothecene mycotoxin deoxynivalenol (DON) in adolescents is higher than in adults [2]. Immunotoxicity is a specific toxicity of concern from mycotoxins. Mycotoxins inhibit the proliferation of leukocytes and suppress the immune system [3]. Trichothecene mycotoxins have the ability to induce apoptosis in macrophages [4]. Other toxins, such as aflatoxin M1 and ochratoxin A (OTA), induce a competitive endogenous RNA regulatory network of intestinal immunosuppression [5]. Moreover, some mycotoxins, including zearalenone and DON, aggravate disease progression by inhibiting the inflammatory response following infection of foodborne bacteria (for example, *L. monocytogenes*) [6] and viruses [7]. On the other hand, the global outbreak of animal diseases is closely related to mycotoxin contamination in the environment, as these toxins reduce the immunity of animals. Moreover, mycotoxin contamination in feed is an important risk factor for animals’ susceptibility to pathogens [8,9].

Currently, studies have focused on the potential mechanisms of mycotoxin immunotoxicity and found that oxidative stress, apoptosis, and autophagy are important toxic effects and mechanisms. Researchers have tried to reveal the upstream regulatory mechanisms from signaling pathways such as hemopoietic cell kinase (HCK) and RNA-activated protein kinase R (PKR) [10,11]. In addition, some crucial regulatory mechanisms have been gradually reported in recent years, such as the TLRs/NF-κB pathway and other crossing pathways, including cyclooxygenase-2 [12], and endoplasmic reticulum stress-activated PERK-eIF2α-ATF4 signaling [13], which play important roles in the immunomodulation of mycotoxins. Moreover, Notch1 signaling is concerned since this pathway is significantly activated after DON exposure [14]. In addition, mycotoxins can inhibit the expression of immune response factors and mediate an intracellular active “immune evasion” process, reducing the body’s cellular immune defense function and altering the cellular immune microenvironment, allowing the toxins to further poison cells [15]. In this regard, the hypoxic microenvironment and hypoxia-inducible factor (HIF) play the unique roles, since HIF-1α inhibits the trichothecene mycotoxin-mediated “immune evasion” process by negatively regulating programmed death-1/programmed death-ligand 1 [16].

Indeed, hypoxia is involved in the toxic mechanisms of mycotoxins [17,18]. In addition to its occurrence in trichothecenes [19,20], HIF-1α is also involved in the regulation of toxic mechanism of other toxins. OTA can upregulate HIF-1α and then lead to tumorigenesis through altering growth signaling transforming growth factor-β and vascular endothelial growth factor [21]. OTA causes kidney toxicity via the AhR-Smad2/3-HIF-1α pathways [22]. In hypoxic conditions, ROS formation is increased in hypoxic conditions, which contribute to further activation of HIF-1α [23]. As a new toxicity mechanism of mycotoxins, the relationship among hypoxia, oxidative stress, and immunotoxicity needs to be studied. Importantly, recent studies further show that cellular senescence plays a potential function in the immunotoxicity of mycotoxins. Cellular senescence can be triggered by oxidative stress in the DNA damage response [24,25,26]. Mycotoxins induce cellular senescence and cause senescence-associated cell cycle arrest [27,28]. OTA increases the senescence biomarkers expression of p53, γ-H2AX, and the senescence-associated secretory phenotype (SASP) inflammatory factors [29]. Moreover, OTA induces renal cell senescence by modulating the expression of cyclin-dependent kinase 2 [30]. Another mycotoxin, alternariol, induces an arrest in the G_2_/M transition by increasing the expression of cyclin B and SA-β-gal activity [31,32]. Aflatoxin B1 (AFB1) upregulates *CXCL8* expression and causes cell cycle arrest [28]. In addition, AFB1 causes G_0_G_1_ cell cycle arrest by downregulating cyclin D1 expression [33]. We believe that other mycotoxins, including trichothecenes, have the capacity of inducing cellular senescence. Actually, in our recent primary experiment, we have observed that trichothecenes, including T-2 toxin, could activate SASP and SA-β-gal and induce RAW264.7 cell senescence, implying that more mycotoxins (including modified ones) have the potential of inducing cellular senescence. Nevertheless, the underlying mechanisms in this context need to be explored. Since cellular senescence and SASP are reported to induce an immunosuppressive environment [34], we suspect that the cell senescence induced by mycotoxins plays a potential role in the immunosuppressive effects (Figure 1). Understanding the function of cellular senescence in immunotoxicity helps to further explore the toxic target of mycotoxins. In addition, whether hypoxia and HIF are involved in this potential mechanism is not yet fully understood, but it should be noted that HIF is a master regulator of the immune evasion process. Other scientific questions, including how mycotoxins induce hypoxia and HIF and what is the molecular mechanism by which cellular senescence regulates immunosuppression, need to be answered in the near future.

Therefore, under these circumstances, we are pleased to have this opportunity to compile a Special Issue entitled “Hypoxia, Cellular Senescence, and Immunosuppression: Emerging Toxic Mechanisms of Mycotoxins and Toxins” for *Toxins* (https://www.mdpi.com/journal/toxins/special_issues/31W44JMZ11, accessed on 16 December 2022). The aim of this Special Issue is to assemble reviews and original research articles on mycotoxins and toxins, including their new toxic mechanisms and signaling and especially on hypoxia, cell senescence, and immunosuppressive effects. We welcome researchers to submit their relevant research articles and excellent work on mycotoxins and toxins to this Special Issue with the aim of sharing the latest ideas and progresses in this field with the readers of *Toxins*.

## Figures and Tables

**Figure 1:**
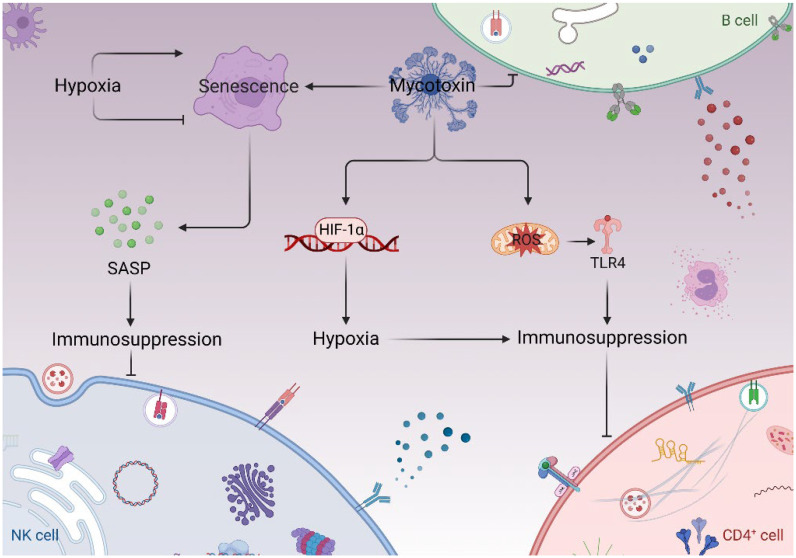
Mycotoxins, hypoxia, cellular senescence, and immunosuppression.
